# Beyond the threshold: real-time use of evidence in practice

**DOI:** 10.1186/1472-6947-13-47

**Published:** 2013-04-15

**Authors:** James B Jones, Walter F Stewart, Jonathan D Darer, Dean F Sittig

**Affiliations:** 1 Geisinger Center for Health Research, Danville, PA, USA; 2 Geisinger Clinic, Danville, PA, USA; 3 University of Texas Health Science Center – Memorial Hermann Center for Healthcare Quality & Safety, School of Biomedical Informatics, Houston, TX, USA

## Abstract

In two landmark reports on Quality and Information Technology, the Institute of Medicine described a 21st century healthcare delivery system that would improve the quality of care while reducing its costs. To achieve the improvements envisioned in these reports, it is necessary to increase the efficiency and effectiveness of the clinical decision support that is delivered to clinicians through electronic health records at the point of care. To make these dramatic improvements will require significant changes to the way in which clinical practice guidelines are developed, incorporated into existing electronic health records (EHR), and integrated into clinicians’ workflow at the point of care. In this paper, we: 1) discuss the challenges associated with translating evidence to practice; 2) consider what it will take to bridge the gap between the current limits to use of CPGs and expectations for their meaningful use at the point of care in practices with EHRs; 3) describe a framework that underlies CDS systems which, if incorporated in the development of CPGs, can be a means to bridge this gap, 4) review the general types and adoption of current CDS systems, and 5) describe how the adoption of EHRs and related technologies will directly influence the content and form of CPGs. Achieving these objectives should result in improvements in the quality and reductions in the cost of healthcare, both of which are necessary to ensure a 21st century delivery system that consistently provides safe and effective care to all patients.

## Introduction

The creation and codification of medical knowledge has grown at a pace that exceeds the ability of health care providers or patients to make effective use of it. Methods for summarizing evidence have advanced, are increasingly standardized, and the infrastructure (e.g., journals, societies, organized teams of experts, etc.) for promoting these endeavors continues to expand. Clinical practice guidelines (CPG) distill evidence as a means to promote adoption of state-of-the-art care. While translation of evidence to CPG has accelerated, especially in the past decade [1], use of guidelines in clinical practice has not kept pace. One of the many reasons for this adoption gap is that, other than learning by traditional means (e.g., CME is proven to be minimally effective) [2], there are few effective non-technological approaches for disseminating evidence to routine practice.

Health information technology can enable routine and automatic adoption of CPGs through tools such as computerized decision support (CDS), but current CPGs are not expressed or formatted for ready use by these tools. There are high hopes that the adoption gap will be addressed by recent legislation [3,4] intended to foster the meaningful use of electronic health records (EHRs) and related technologies such as CDS. In this paper, we: 1) discuss the challenges associated with translating evidence to practice; 2) consider what it will take to bridge the gap between the current limits to use of CPGs and expectations for their meaningful use at the point of care in practices with EHRs; 3) describe a framework that underlies CDS systems which, if incorporated in the development of CPGs, can be a means to bridge this gap, 4) review the general types and adoption of current CDS systems, and 5) describe how the adoption of EHRs and related technologies will directly influence the content and form of CPGs.

## The challenge of translating CPGs to practice

There are over 2,000 guidelines in the national guideline clearinghouse [5]. It is widely recognized that adoption (i.e., application of the guideline to a specific patient at the point of care) of these guidelines is limited [6]. The volume of knowledge even for a single clinical area is daunting. For example, the guideline for asthma care [7] is over 400 pages long and its recommendations are based on the need for data (e.g., pulmonary function testing, symptoms) that physicians do not routinely collect in codified form from patients, even in an EHR-based setting. The gap between the creation of evidence and its use in practice has largely contributed to a dramatic growth in the volume of best practices “parked” at the threshold of the clinical practice setting [8,9]. The evidence is clear that the century-old health care education and delivery model will not keep pace [10].

There are inherent barriers to translating CPGs to practice. CPGs are typically promulgated in lengthy documents of written prose or as graphical displays (e.g., decision trees or flow charts), are often ambiguous and non-committal, use highly variable non-standard forms of medication, laboratory test, and procedure names, are largely inaccessible for practical purposes, and, in the absence of the ability to translate CPGs to a structured form of data, are often totally inaccessible via computer applications (except as free text displays) [11]. Often the prose or graphical images within a CPG cannot be easily or reliably translated to logical, operational rules. Even if well-specified operational rules were available, it would be practically impossible for most providers to routinely learn and use CPGs in routine practice.

Learning a CPG represents only one step in the effective use of knowledge [12]. The time that is available to review, test, internalize, and accurately apply CPGs is limited in clinical practice. Additional time must be invested by a physician to process, internalize, adopt, and eventually use a CPG in practice. Continuing education, the dominant method by which physicians formally augment their knowledge, is largely a peripheral activity for physicians and the mode of learning is divorced from clinical practice. Evidence consistently demonstrates that current methods of education have limited impact, at best, on quality of care [13]. The growing adoption of EHRs and other forms of HIT affords a unique opportunity to explore how continuing education can be seamlessly integrated with the daily routine of care delivery to address fundamental challenges with effective use of knowledge [13]. However, even if one were able to keep pace with advances in knowledge, the application of this knowledge at the point of care will still be extremely difficult without some form of cognitive aid [14]. For example, guidelines must be applied to patient-specific data to be useful. Often, the data required to assess eligibility of a particular patient for a given guideline or to determine which of the many different treatment options is applicable to a specific patient is either not available at the point of care or would require too much time to ascertain in a useful form during an encounter.

The challenges of translating knowledge into practice parallel those that have plagued other information-rich service sectors; the ways in which other sectors have overcome these challenges have implications for health care. Information-rich service sectors are those in which the volume of knowledge and data required to deliver state of the art services requires a systematic and integrated translation process and automated and machine-enabled human interactions. For example, financial planning was once dominated by a paternalistic service model. High-quality advice and information were available through “knowledgeable experts”, primarily to those who could pay. With the transition from defined-benefit to defined-contribution pension plans, the consumer-focused market emerged, supported by the availability of sophisticated web-based tools and widespread access to data and information. Consumers began to assume a more active role in their own financial planning. While consumers can and do make irrational decisions in this role, they have the option of being guided by sophisticated, easy-to-use programs (e.g., risk profiling tools that map to fund allocations and automated age-based asset rebalancing, etc.), that narrow the knowledge gap between the consumer and an investment professional. Consumers now have access to high quality information, online tools and back-up human support; while the business seeks to influence the selection of an “optimal” choice, it doesn’t feel “responsible” for the consumers’ ultimate choice. The fundamental shift in the financial planning sector has been motivated by the systematic application of knowledge to data combined with tools that allow consumers to access such information in a manner that is tailored to their individual needs.

Health care information and service is considerably more complex than financial planning. Yet, there are general parallels with regard to patient and consumer information needs (e.g., access to knowledge, evidence on risks and benefits, personal data including preferences for risks and benefits, rules applied to data), and important, well-understood differences (e.g., nature of the markets, complexity, lexicon, regulations, legal risks, operational aspects of services, role of human compassion and understanding) between health and financial management. Notably, health knowledge and the data required to use such knowledge are inordinately complex, making it difficult, if not impossible, to completely separate consumer use of information from the need for a trusted relationship with a provider; consumers can manage their own investment portfolios, but they are unlikely to become their own doctors. However, similar to the changes wrought in the investment service sector, a broad-based solution to making health care knowledge translatable begins with the process by which knowledge is assembled for use in health care.

**Figure 1:**
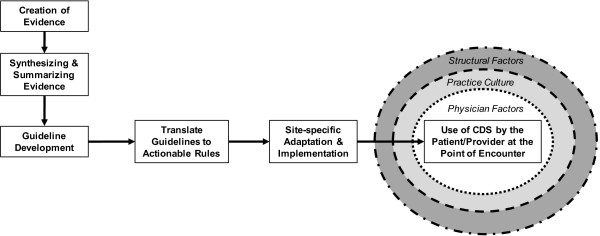
Virtual integration of major steps in translation of clinical evidence to use at the point of encounter.

In health care, there are countless independent groups and entities involved in the creation of evidence and the translation of evidence to practice, albeit, in a non-systematic manner and with conflicting recommendations and lack of integration or harmonization in the same clinical domain. The lack of a conceptual model for a systematic and integrated translation “process,” will continue to ensure that each domain of activity relevant to bringing knowledge to practice will function somewhat independently and continue the ongoing “warehousing”, rather than the effective use, of CPGs. The medical knowledge translation enterprise is unique in comparison to other types of industries in which seemingly independent groups naturally work together in bringing products and services to customers. In medicine, by contrast, groups of individuals work independently of each other without the mission and vision of a larger purpose to ensure that providers and patients can routinely access the knowledge that they need and want. Transformation of the current “process” will largely depend on the virtual integration of many different independent activities, where the notion of integration is guided by how to effectively bring and use knowledge at the point of care (Figure 1). The implications of virtual integration for knowledge translation are as important for what will be required in codifying knowledge as they are for what will be required in the clinical practice setting to make effective use of such knowledge. The last step in the translation process involves the adoption and meaningful use of information technology. This step is fraught with complexity and the influence of, and socio-technical interactions among, physician factors, practice culture, structural factors, and patient factors [15]. Indeed, because each patient is unique, this last step will always require physician judgment about the applicability of the evidence to an individual patient and his/her clinical scenario. While we acknowledge the importance of these factors, we confine our discussion to how CPGs may be developed so that they can be more easily used in clinical settings with EHRs.

In part, virtual integration will be motivated by the accelerated adoption and meaningful use of EHRs and other forms of information technology in clinical practice. This is not to say that the use of the EHR itself will lead to integration. Rather, there have been and will continue to be upstream effects on codifying knowledge that are influenced by those who develop clinical decision support and quality measurement protocols. For example, the desire to perform drug-allergy interaction checking has prompted the need for providers to accurately enter their patients’ allergies, reactions, and severities using a standard clinical vocabulary and to accurately maintain active/current medication lists.

The translation of CPGs to clinical practices with EHRs will strongly depend on the use of CDS. In fact, current CDSs are often the product of translating guidelines to computer code and operationalizing the process, including integration with clinical workflow. The initial implementation of an EHR is often rapidly followed by naïve attempts to implement and use rudimentary forms of CDS (e.g., a hard stop for drug-drug interaction alerts). More robust forms of CDS, however, require the translation of “knowledge” (e.g., as embodied by guidelines) to a structured form before it can be used in an EHR CDS protocol. Despite numerous attempts, to date, there is no universally accepted format for translating guidelines into CDS-related protocols to facilitate adoption. CDS interventions are usually idiosyncratic to a given health care setting with an EHR, are rudimentary, and are often interruptive, unhelpful, and unsatisfying to providers. The lack of well-accepted standards for clinical vocabularies, CDS formats, clinical workflow application, and lack of clinical and patient-reported data in accessible, codified fields, currently limit the ready use of CPGs [16,17]. In this White paper, we consider what virtual standardization and integration of the knowledge into practice will mean in an era where EHRs are widely used. We first describe a functional taxonomic framework which characterizes how the CDS process works in routine care and consider the implications of this framework for creation of actionable CPGs.

## General CDS framework

CDS systems have been developed in a variety of forms to serve a diversity of functions [18]. A common framework can be used to characterize those forms of CDS that involve interactive, point-of-care interventions and may be helpful in revealing one method in which CPGs can be structured to be more actionable. This type of interactive CDS relies on structured patient *data* as the key input that are processed by knowledge based *rules, or statistical algorithms* to generate an *output*[19,20]. While the desired CDS process will vary depending on the objective, context, and available patient data, the depth, form and quality of the data and the CDS rules will dictate the limits of what is possible with the output (i.e., from simple generic alerts to intuitive and tailored visual displays of information). While the above framework is standard, there is no universally accepted format for translating guidelines into this framework for widespread adoption and use, although several commercial vendors have approved and adopted an HL7 standard called the Arden Syntax for medical logic modules [21,22].

Wright et al. offer a taxonomy for interactive, point-of-care CDS comprised of four functional features: 1) *triggers*, or the events that cause decision support rules to be invoked (for example, prescribing a drug); 2) *input data* elements used by a rule to make patient inferences; 3) *interventions*, or the possible actions a decision support module can take; and 4) *offered choices*, or the options available to a decision support user when a rule is invoked (for example, change a medication order) [23]. We describe these functional features and their implications for an optimally-designed CDS process for integrating with CPGs.

The *trigger* is the initiating step in the CDS process. The patient data that are available in real time will vary by clinical setting and other factors, such as the type of EHR in use or decision to use free text versus discrete data fields. The utility of CDS systems can be optimized by recognizing this variability, defining standards for minimal and optimal data inputs, and offering meaningful utility at both ends of the data availability spectrum. Moreover, data that are used in the triggering process should be based on a reference standard for a given data domain, such as RxNorm for drug names [24], SNOMED-CT for clinical problems [25], and LOINC for laboratory tests [26].

The *input data* are fundamental to deploying CDS that it is relevant to the right person, with the right information, and output in the right format, features deemed critical to optimizing CDS [20]. Medication orders, laboratory data, problem list and encounter diagnoses codes, and administrative data, for example, can all serve as inputs to a rule process used to generate decision support outputs. In some cases, input data will be poorly represented in an EHR system. For example, the USPSTF guideline for gonorrhea requires an assessment of sexual activity, input data that may not be routinely recorded in a structured format within an EHR (although virtually all EHRs have the capability of recording this information in a coded form, e.g. via the social history tab in the EHR). Theoretically, input data can be obtained directly from patients. Historically, the collection of patient-reported data (PRD) in routine practice has been limited by the operational and logistical challenges associated therewith [27]. Without actionable patient data, the key steps in the translation process (Table 1) are unlikely to occur in a seamless and automated manner. The emergence of web-based technologies will allow for the capture and real time use of structured PRD. However, PRD will not be useful in facilitating translation of CPGs to practice unless data are captured in a reliable, accurate, and actionable form to represent patient experience and can be mapped to existing clinical vocabularies.

**Table 1:** Translating knowledge for use at the point of encounter: stakeholders and challenges to integration

**Step**	**Responsible stakeholder(s)**	**Challenges to integration**
Creation of evidence	Researchers in academic medical centers funded by NIH, AHRQ etc.; industry funded RCTs; Foundation funded initiatives	RCT evidence is often limited in how it can be generalized for use in routine clinical practice; everyday clinical questions, especially for multi-morbid patients are not specifically addressed. The lack of comparative effectiveness data limits utility of existing evidence
Synthesizing and ummarizing evidence	Medical societies, health systems, clinical content vendors	Synthesis and summary are foci for this process, not application and actionability; many clinical actions do not have sufficient RCT evidence for action
Translate evidence for use by EHRs	Health systems, health information technology and clinical content companies, software companies	No established standards to operationalize alerts, order sets, documentation templates, or hyperlinks to content to facilitate the use and delivery of CPGs; lack of knowledge of effectiveness of computer-based intervention options and of meaningful use of HIT
Site-specific adaptation & implementation	IT staff, providers at clinics with EHRs	Adapt to local workflow, policies, best practices; map content to local nomenclature or orderable catalogs
Use at the point of encounter	Providers	Changing physician behavior; accurate identification of exceptions; overwhelming number of non-specific recommendations
Evaluation of the effect of the evidence as implemented on patient outcomes	Quality assurance, risk management, or organizational administrative departments	No standard way to identify patients for either the numerator or denominator of the measures; many key data items not available in coded portion of the EHR; current quality measures not linked to CDS interventions.

The *intervention* refers to the possible actions such as activating a passive or active physician alert, displaying relevant information, or displaying a relevant guideline with supporting patient data. An optimal CDS model should allow knowledge engineers to specify the criteria that govern which interventions are available and the rules that govern the interplay among a trigger, input data, and the intervention [28]. For example, the previously mentioned gonorrhea rule might be triggered for all male patients within a certain age range, but the intervention may be a passive reminder when the patient’s sexual activity is not known versus an active alert when all patient data are readily available. This flexibility in specifying *interventions* based on *input data* and *triggers* is critical; physicians may be less likely to use an alert if it does not specify an action, specifies a generic action, or specifies one that is incongruent with the input data [29-31]. CDS will be perceived as more useful if it reduces work demand and less useful if it creates unnecessary demands (e.g., more clicks to order the optimal medication). However, designing CDS protocols in this manner is challenging because of the diversity of treatment management scenarios for a given clinical domain. We have explored such challenges at Geisinger in developing an EHR-based CDS model (“eDiabetes”) for expert treatment guidance and management of HbA1c in diabetes. Four input variables are used to identify relevant treatment advice among 93 distinct possible messages. Notably, each additional input variable increases the specificity of the advice that can be offered, but exponentially increases the size of the CDS database and the challenges in maintaining the knowledge base, rules set, and veracity of the output [32].

*Offered choices* are the options that can follow the result of a notification *intervention*. For example, at each office visit a rule may be *triggered* to evaluate a patient’s low-density lipoprotein level; if the level is elevated, an alert may be evoked to notify the physician to prescribe a statin. The *offered choices* may then include the option to write a medication order, defer the alert, schedule a re-test, or add a new diagnosis to the problem list, among others [33]. Alternatively, the rule might also check if a statin has been ordered in the past to increase the specificity of the offered choices. The range of options will vary based on the clinical setting, extant workflows, the end user (nurse or physician), available technologies, and the availability of *input* data. An optimal CDS model must be sufficiently flexible to accommodate a diversity of *offered choices* given the diversity in the other three functions.

CDS is likely to evolve rapidly over the next decade, but various forms of CDS are likely to involve the above-described features regardless of the level of sophistication. For CPGs to be more actionable in a digital environment, their structure will, to a significant degree, need to mirror this CDS structure. Specifically, CPGs will be more useful if they are structured to define the relevant patient subgroup and/or data (i.e., the triggers and input data), the intervention options, and the offered choices that will guide both the physician and patient in making optimal, evidence-based decisions.

## Types and forms of current CDS tools

CDS tools, which are largely based on the translation of CPGs, can be used for diagnostic decision support, preventive care reminders, disease management or protocols for bundles of reminders, and drug dosing/prescribing protocols, among other less common applications [34].

In describing evidence on the effectiveness of forms of CDS, we also consider the likely evolution of CDS protocols. A common view is that future CDS protocols should facilitate the delivery of “the right care to the right person at the right time” [35,36], representing a more personalized and timely form of guideline based care. Ultimately, the utility of a CDS protocol will be first judged by how often it is actually used when intended and, if used, whether the protocol improves processes of care and patient outcomes. Given the lack of knowledge about CDS protocols, the lack of standards, and the current state-of-the-art, evaluating the comparative effectiveness of CDS protocols will be confounded by numerous factors including the extent of integration with extant workflows, physician demand to make use of the CDS (e.g., choosing and ordering offered choices), the quality of the advice delivery mechanism (e.g., reminder versus patient tailored treatment guidance), and the face validity of the process itself. With regard to these factors, Table 2 summarizes representative CDS applications.

**Table 2:** Examples of currently deployed CDS tools and their incorporation of CDS factors

**CDS type**	**Goal of CDS**	**CDS specificity**	***CDS elements***			
			**Trigger**	**Input data**	**Intervention**	**Offered choices**
Diabetes electronic management system [37]	Improve care of patients with diabetes	Generic	Diabetic patient presenting in clinic	-	Care prompts based on ADA guidelines; some tailoring based on user (nurse vs. doc vs. diabetes educator	-
		Highly tailored	-	Accepts data from institutional data systems		Printed AVS for patient; timing of next visit, tests, referrals can be indicated and printed in a document for administrative use (follow-up)
Osteoporosis CDS [38]	Deliver patient-specific guideline advice to primary care physician via EHR message	Generic	Search of electronic databases for patients meeting criteria for increased osteoporosis risk		Tailored inbox message in EHR that links to patient record	Inbox message lists internal and external guideline resources that provide detailed information on osteoporosis evaluation and management
		Highly-tailored		Demographic and diagnostic information from the EHR used to identify patients requiring management		
Academic information platform for CPG Use in practice [39]	Improve guideline- recommended osteoporosis care using EHR reminders	Generic	Physician volition (i.e., no EHR-based trigger)	None	Availability of Web-based or CD-ROM based access to text of guidelines for dementia, CHF, UTI, and colorectal carcinoma	None; guidelines are read-only
Internet-based decision support for tuberculosis therapy [40]	Improve physician knowledge of guidelines	Generic	Physician volition (i.e., no EHR-based trigger)	Physician-provided data on patient characteristics and clinical reaction to diagnostic test	Web-based implementation of hierarchical decision tree for administering preventive therapy	Guideline-based recommendations for treatment
Clinical reminders for diabetes, coronary heart disease [41]	Improve quality of care for diabetes and heart disease using EHR reminders	Generic	-	-	-	Care recommendation; reminders were actionable but did not require acknowledgement or link to intervention
		Highly-tailored	Physician opens medical record	EHR data (lab, radiology results, problem list, medication list, allergy list)	Reminders list in the EHR in the context of other patient data	-
Asthmacritic [42]	Provide patient-specific asthma treatment feedback using EHR data	Generic	-	-	-	-
		Highly-tailored	Automatic when record is open and asthma-specific data is entered	Physician-entered data on diagnosis and treatment	On-screen patient-specific comments presented to physician, tailored to current clinical situation	Physician presented with “critiquing comments” related to treatment decisions; can drill down to view guidelines to understand reason for comment
Respiratory CDS [43]	Improve standardization and quality of ventilatory care	Generic	Patient “enrolled” in protocol-based care, then driven by arterial blood gas results		Order suggestions displayed to clinicians on bedside computer terminals	Clinician can document acceptance or rejection of the computer-generated suggestion
		Highly-tailored	Arterial PO_2_ < 60 mmHG	Respiratory therapist charting, many other data items used (radiology results, vital signs, etc.		Clinician can order increase in F_i_O_2_ or 10%, followed by an arterial blood gas in 15 minutes

### Diagnostic decision support

Diagnostic decision support systems (DDSS) [44] represent one of the earliest forms of CDS innovations [45,46]. DDSS relies on clinician or patient input of relevant data (e.g., signs, symptoms, laboratory values) that are processed by a knowledge base to return potential diagnoses. DDSS systems are often as accurate as clinical experts in making a diagnosis, but are not used in practice [47] and have not been successful in consistently improving outcomes [34]. One reason for the apparent lack of use may be the workflow constraints to obtaining the volume of data in the right format required for an accurate diagnosis [48]. In addition, few of these systems provide guidance on treatment once the diagnosis is determined (no offered choices). From a functional standpoint, DDSSs will have limited utility in routine primary care settings unless they are integrated with other CDS protocols (e.g., recommendations for medication orders).

### Reminder/alert systems

Preventive care reminders represent another of the early and most common forms of decision support [49] that is common to most EHRs and particularly focused on preventive care [16]. Alerting protocols are highly heterogeneous and evidence on effectiveness is mixed; they have been shown to improve preventive care [50], but multiple studies have also found high rates of overriding alerts and reminders in physician order entry and decision support systems [29].

Point-of-care computer reminders, a rudimentary form of decision support, can improve effective use of care processes (i.e., alerts for prescription orders, recommended vaccine, test order, clinical documentation) and avoidance of unnecessary care. The median effect of tested forms of alerting (i.e., <10%) when compared to usual care, however, is well below a clinically meaningful threshold for even process measures, let alone patient outcomes [51]. Poorly designed alerts (e.g., too frequent, insufficiently specific, workflow-impeding, etc.) can lead to “alert fatigue”, where physicians ignore both important and unimportant alerts [52]. “Alert fatigue” (leading to ignored alerts, as well as an increased propensity to ignore future alerts) is a side effect of the rapid growth in deployment of alerts, especially protocols that are non-specific, poorly targeted, direct the provider to take action, and, more generally, low in clinical content [29].

### Diagnostic imaging

Diagnostic imaging CDS (DI-CDS) offers guidance on appropriate use of imaging procedures for diagnostic purposes. Notably, there is relatively little observational or RCT evidence on effectiveness of imaging. In addition to identifying redundant orders, almost all guidance is based on expert opinion. Diagnostic imaging CDS systems make use of a utility score for a given care scenario. The score is based on the American College of Radiology Appropriateness Criteria [53]. A low score does not necessarily prevent an image order. Physician overrides require documentation that can be used to refine future CDS algorithms. Diagnostic imaging CDS systems interface with EHRs and computerized physician order entry (CPOE) systems to enable physicians to place diagnostic imaging requests as usual, but with the presentation of advice if and when alternative tests should be considered. When a request receives a low score relevant decision support is provided and a request for additional physician entered data may be required. These CDS tools represent an important advance in solving data standard and technical interface challenges, and their adoption is likely to accelerate [54]. For example, state legislatures (e.g., Minnesota, Washington) have or are considering mandating use of imaging CDS [55,56]. While diagnostic imaging CDS is sometimes viewed as a cost reduction substitute to insurance-mandated prior authorization, management of inappropriate use of diagnostic imaging has potentially significant safety implications (e.g., reducing unnecessary surgery, reducing radiation exposure from CT).

### Drug dosing and prescribing

Drug dosing and prescribing systems are designed to guide the selection of a therapeutic agent for a given clinical scenario and to select an optimal dose [57]. Drug-based CDS may also provide warnings about potentially dangerous drug combinations (i.e., drug-drug interactions) [58]. In a review by Garg *et al.,* drug-based CDS improved provider prescribing performance in the majority of evaluated studies, but with minimal impact on patient outcomes [34]. Many of the studies of drug-based CDS focused on a narrow set of medications or conditions (e.g., anticoagulation). Electronic ambulatory care prescribing (eRx) systems are becoming increasingly common tools for automating the medication ordering process across a wide variety of conditions and medications. eRx systems may be integrated into an EHR or be stand-alone applications. A systematic review of 27 studies of electronic prescribing found that half of the included systems had advanced decision support capabilities (e.g., contraindications, allergy checking, checking medication against laboratory results, etc.) in addition to the medication ordering function [59]. Although there is evidence that electronic prescribing can reduce medication errors and adverse drug events, the evidence is limited, particularly in outpatient settings [59]. The inclusion of eRx in the meaningful use requirements set forth by the Office of the National Coordinator for Health Information Technology (which will impact Medicare reimbursement rates) is likely to accelerate research in this area [60].

### Condition- or task-specific clinical documentation and order entry forms

Structured data entry is essential for all forms of interactive, clinical decision support, as well as the most difficult aspect of EHRs for clinicians to adopt. In an attempt to capture accurate, coded, clinical data, EHR designers and developers have developed interface terminologies [61], condition- or task-specific clinical documentation [62], and order entry templates or forms [63]. These forms have been shown to improve the quality of patient care documentation as well as outcomes in limited study [64].

Finally, there are a variety of different forms of CDS that present high quality evidence –based forms of CDS. For example, UpToDate, Micromedex, CliniConsult, ClineGuide, etc. are common forms of clinical decision support that provide clinical knowledge at the point of care [65]. In general, while these examples offer the dominant means by which most providers access clinical guidelines at the point of care, most of these implementations lack the essential patient- or condition-specific features of the previously described CDS framework and are substantially less actionable. There is work underway to develop a standard method of accessing context-specific information that exists in a computer system external to the main clinical information system called the “Infobutton” that offers great promise [66].

## Current state of CPG and CDS adoption

### CPG adoption

CPG adoption, like clinical care, is not a simple binary process (i.e., adoptions occurred or did not). Rather CPG adoption encompasses implicit and explicit elements including awareness (clinicians must know that a guideline exits), evaluation (i.e., clinicians must assess the applicability of a guideline to a specific patient), obtaining and reviewing data, interpreting data, and adherence (physician actually follows the guideline). Studies that do not measure implicit steps (e.g., awareness and evaluation) may falsely conclude that a CPG was not adopted, despite the fact that a physician may consult a guideline (awareness and evaluation) and ultimately decide that it does not apply and so the recommendation is not followed. An evaluation that focuses only on overt adherence will fail to acknowledge that the guideline was used appropriately.

Awareness of a guideline is one step in a process towards guideline adoption [6]. McGlynn (2003) found that patients receive guideline recommended care only about 50% of the time [8]. Improvements in outcome measures have been noted in condition-specific studies [6]. It is unclear whether these findings generalize across a diversity of conditions.

### CDS adoption

The relatively poor adoption of CPGs in practice is partly due to the lack of an effective workflow model that will enable efficient use. Over the past 30 years, a variety of CDS systems have been developed and evaluated. Although benefits have been demonstrated, many of these implementations are unique to a single system, confined to use in an academic medical center that has had a long standing working relationship with the developers, or rely on increasingly outdated (e.g., use of paper printouts that are attached to the chart) approaches to delivering recommendations [67].

The increasing adoption of electronic health records (EHR) will result in increased physician exposure to rudimentary forms of CDS and possibly more advanced forms. The ONC’s recently-released final rule on EHR certification requires decision support functionality, as do the meaningful use criteria that are designed to drive EHR adoption [4,68]. Less than ten percent of U.S. hospitals have a basic EHR system and less than two percent have a comprehensive EHR system [69]. Recent evidence indicates that only about 17% of outpatient practices use at least a basic EHR system [70].

Chaudhry et al. found that approximately 25% of all English-language peer-reviewed studies that have been used to demonstrate increases in quality and safety in patient care have originated from four institutions [67], each of which had internally-developed EHRs with advanced CDS features and functions and long-standing collaborations with the users and developers of the EHR and CDS. More recently, a growing number of large integrated delivery systems (e.g., Geisinger, GroupHealth Cooperative of Puget Sound, Kaiser Permanente) have adopted commercially-developed multifunctional health information systems. Despite burgeoning adoption, there is little evidence regarding the effectiveness of these commercially-developed systems [67,71].

## CDS and CPGs in the future

The adoption and meaningful use of EHRs and HIT has the potential to transform the pace, specificity, quality, and utility of how knowledge is translated into practice. To this end, it will be important to translate evidence into a reliable, valid, and structured form so that it can be more readily used with HIT in a manner that is useful to providers and patients. Recent legislation will foster and compel adoption and “meaningful” use of EHRs to prime the change process. However, one of the dominant concerns in motivating adoption of EHRs among physicians, let alone meaningful use, is the cost and utility of technology [70]. Thus, it is likely that sustainable transformation will depend on how effectively and efficiently new technologies help providers achieve outcomes they each deem to be a priority and that improve efficiency of care in ways that also improve the quality of care. We consider how more structured CPGs will be important to adoption and meaningful use of EHRs.

The growing use of HIT in clinical care represents a fundamental shift. The notion of “meaningful use,” a recent addition to the HIT lexicon, is itself an indication of the shift that is underway. The adoption of HIT alone will not be sufficient. The Health Information for Economic and Clinical Health (HITECH) Act is intended to foster adoption of HIT and its meaningful use through financial incentives and with increasingly demanding reporting requirements that will be linked to reimbursement rates [72]. The definition of meaningful use is expected to evolve over time as the utility and capabilities of HIT improves. Currently, the definition emphasizes the electronic capture of coded health information, use of information to track key clinical conditions, communication of information for care coordination purposes, and initial reporting of clinical quality measures and public health information. A “meaningful user” will be evaluated in relation to a core set of objectives and related measures [73]. In 2013, the definition is expected to encompass data and process needs relevant to disease management, clinical decision support, medication management, patient access to their health information, transitions in care, and quality measurement and research. In 2015, the definition is expected to further expand to include a focus on improvements in quality, safety and efficiency, decision support for national high priority conditions, patient access to self management tools, access to comprehensive patient data, and improving population health outcomes. Although the financial incentives (and disincentives) forthcoming as part of the HITECH legislation are intended to drive adoption of EHR, truly “meaningful use” will depend on many socio-technical factors, including those outside the scope of this review (e.g., culture, finance, physician preferences, etc.) [74]. The effective translation of knowledge to practice will depend, in part, on what we know about computerized CDS and its impact on care processes and patient outcomes, factors that are central to advancing the vision inherent to HITECH.

Alerting systems provide a cautionary tale about the importance of designing systems that are likely to be “meaningfully used”. Currently, RCTs indicate that alerts fail on the most basic measure of utility. Physicians often ignore alerts and alert-aided advice (even though the advice is evidence-based); this strongly suggests that physicians do not perceive these “aids” as useful. Certainly, clinic culture and physician attitudes are important to motivating adoption of methods known to improve outcomes [75]. It is easy to blame the intended audience for not cooperating and overlook other factors. The proximal cause of an alert failure may be that it is poorly designed, poorly timed, designed for someone other than a physician (e.g., quality managers), or directed to the wrong person, factors that have a bearing on the structure and related utility of CPGs [76].

While simple reminder alerts directed to physicians have minimal impact, there is also little evidence to suggest that more sophisticated forms of CDS improve care processes and patient outcomes. Again, however, the lack of support may simply reflect flaws in how CDS processes are designed (i.e., do they address needs of the end user). As previously noted, structured CPGs are intimately linked to data inputs. More structured CPGs that specify the required content and format of data and the skill level required to manage the CPG will support more sophisticated means of providing guidance to the end user.

While alerts are increasingly being evaluated in randomized controlled trials, we know relatively little about the diversity of alerts actually used in clinical practice and the factors that govern the effectiveness of such alerts. Moreover, while RCT evidence serves as a gold standard, many important questions about CPGs and CDS will not be addressed by RCTs. The time and cost required to initiate RCTs limits the diversity of questions that can be answered. Observational data may also be valuable in providing guidance on what forms of CDS do and do not work [77]. In particular, a growing number of integrated health care systems are using ambulatory and inpatient EHRs. There is intense interest in using the longitudinal clinical data from these systems for comparative effectiveness research [78]. Relatively little has been said about the diversity of CDS protocols used by such systems and the ability to link clinical care process and outcome measures to EHR log files that contain time stamped-data on CDS transactions, including the encounter during which an alert was presented, whether the alert was accessed (i.e., if it is not a hard stop or a simple display of information), how long the alert remained unacknowledged, etc [79]. These data offer a potentially valuable source of comparative effectiveness evidence on the effectiveness of various forms of CDS that are actually used in clinical practice and may be helpful in rapidly advancing understanding of what forms of CDS do and do not work.

CDS should be designed to serve the needs of the end user. Uni-dimensional or binary alerts designed to get physicians to do something or avoid doing something fall short for a number of reasons. Physicians are trained to do cognitively demanding tasks, to process complex information, and to make judgments in the face of uncertainty. Accordingly, they may not be effective or efficient in performing rudimentary tasks that are better suited for automation or completion by a less-skilled individual. Physicians also face demands to be more productive. This is not to say that simple alerts and reminders are not potentially useful to improve care processes and outcomes. Rather, forms of CDS should be hierarchically defined based on the specificity of the CPG, the complexity and specificity of the data required for deployment, the risks associated with various options, and patient preferences (Table 3). Together, these features will likely dictate the optimal timing of deployment in the care process, the optimal decision-maker(s) (i.e., physician, nurse, patient), and the utility of other forms of support (e.g., linked order sets) that are integral to the CDS to engage the end user. In this context, it will be important to consider how CPGs can be structured to allow physicians to do tasks that they could not do otherwise, or that help them to do tasks better and more efficiently.

**Table 3:** Simple and complex forms of CDS

**CDS complexity**	**Example**	**Data needs**	**Risk from interventions**	**Patient preference**	**Clinical decision-makers**
Simple	Routine HbA1c Testing for diabetes	Lab results	Very Low	NA	Automate
Moderate	Hypertension management	BP, patient preferences, engagement, adherence	Low	Prioritizing options	Nurse
Complex	Depression management	Symptoms, chronicity, work role,	Low	Preferences for side effects, use of meds	Physicians Assistant
Very Complex	Chronic low back pain & depression	Signs & symptoms, psychosocial factors,	Moderate to high	Preferences for side effects, use of meds	Physician
Very Complex	Prostate cancer management	Pathology, labs, signs & symptoms, patient preferences	High	Prioritizing needs, risk avoidance	Physician
Very Complex	Ventilator management	ABGs, respiratory therapy, radiology, cardiovascular monitoring	High	NA	Physician decides on protocol, but RT or RN initiates therapeutic changes

The changes that are occurring in large delivery systems indicate that use of EHRs including CDS is changing work roles [80]. These changes have implications for CPGs and the need to articulate the skill level required for a given task. Moreover, where there is strong evidence for when and for whom a care process or treatment (e.g., pneumovax in older patients) should be done, it may be sensible to simply automate the task so that it occurs 100% of the time [81]. For example, all Type-II diabetics without a recent HbA1c should have this laboratory test completed at appropriate intervals, but neither the decision nor the completion of the test requires the involvement of the physician. In this example, CPGs could be structured to recommend a link between the HbA1c level, the importance of other covariates (e.g., liver function tests, kidney function test), who should manage the patient (i.e., nurse, primary care physician, endocrinologist), and the ongoing need for care (i.e., automate decision about next scheduled visit). Thus, where evidence about what to do is robust and understandable (e.g., management of hypertension, hyperlipidemia, etc.), risks are low, and within the limits of common sense, as much control as possible should be shifted to others. Where the risk of confusion and of making the “wrong decision” increase (e.g., as decision complexity increases), decision support tools may become increasingly important and useful for both the provider and others involved in the care processes. In addition, patient guidance and preferences are likely to be important in a shared decision approach to care. Future development of CPGs may consider the extent to which care processes and decisions can be assumed by others and where patient preference is important.

Depending on the risks, strength of evidence, complexity of the decision and intervention, and role of patient preferences, CPGs should designate the end users and also be structured with the end user in mind (i.e., patients, administrative staff, mid-level providers, or physicians). Changes in work roles will also affect the physician. Less time will likely be spent on routine care decisions that can be semi-automated or managed by others. More time will be spent on collaborative care and cognitively demanding care decisions.

The increased adoption of EHRs will open unique opportunities to move clinical knowledge beyond the threshold of clinical practices. However, it will be difficult to fulfill the vision for meaningful use without making substantial advances in standardization and codification of CPGs such that they can be more uniformly adopted across diverse clinical care settings.

In our view, the intersection of CPGs and CDS should be an area of active research to foster the development of actionable forms of CPGs. The AHRQ-funded CDS Consortium (CDSC) has studied CDS practices at five different institutions with both commercially-developed and internally-developed EHR and CDS systems; the goal of this effort was to develop recommendations for CPG development activities that complement and build upon existing knowledge and systems. We support the seven focused recommendations that highlight the interdependence between CPGs and CDS development to achieving the vision of the “digital future” [17]. We summarize these recommendations in Table 4.

**Table 4:** CDSC recommendations for CPG development activities

**#**	**Recommendation**	**Description**	**Rationale**
1	Identify standard data triggers	Guidelines should explicitly identify clinical or administrative data required to initiate any of the CDS interventions included in the guideline	Required data need to be captured and stored in structured and coded fields in order to be utilized by CDS systems
2	Review Access to Existing Input Data	Commonly available input data for use by CDS logic (e.g., for alerts) include: laboratory test results, patient demographics, and the problem list. CPGs should specify only specify coded data types which are currently or soon will be available in certified EHRs	Input data that are not available in certified EHRs will results in guidelines that cannot be incorporated in a computable manner within EHRs
3	Work on increasing clarity and internal consistency of all clinical logic included in guidelines.	CPGs should minimize the ambiguity of their recommendations (e.g.,include threshold values for blood pressure rather than stating “if the patient’s blood pressure is high then…” ).	Logic in CPGs must be able to be incorporated in a computer executable form
4	Suggest appropriate personnel and best insertion points in the clinical workflow for CDS interventions to be delivered	CPGs should specify how the EHR can route recommend actions to the appropriate person or role, at the right time and in the right place, based on logic included with the CDS intervention	Increase CDS utility, efficiency, and integration with clinic workflows
5	Guidelines should facilitate selective filtering or tailoring of rules	Specify explicitly when particular rules either apply or don’t apply in the rule’s logic description.	Allow rules to be turned off when they do not apply to a clinical context (e.g., specific practices, physicians, specialties, or clinical situations)
6	Guidelines should support the HL7 Infobutton standard	Specific definitions of items such as clinical problems, medications, and laboratory tests should be clearly defined using standardized data types	Allows EHRs to link to specific sections of a guideline and provide context-sensitive explanations
7	Composition of guideline development groups	CPG development groups/committees should include well-trained and experienced clinical informaticians	CPGs will be easier to transform into computer executable forms

## Summary

If we are to realize the 21st century healthcare delivery system called for in the Institute of Medicine’s landmark reports on Quality and Information technology [82,83], and reduce the astronomical costs associated with this care, we must increase the efficiency and effectiveness of the clinical decision support that is delivered to clinicians through electronic health records at the point of care. To make these dramatic improvements will require significant changes to the way in which clinical practice guidelines are developed, incorporated into existing EHRs, and integrated into clinicians’ workflow at the point of care. Achieving these objectives should result in improvements in the quality and reductions in the cost of healthcare, both of which are necessary to ensure a 21st century delivery system that consistently provides safe and effective care to all patients.

## Pre-publication history

The pre-publication history for this paper can be accessed here:

http://www.biomedcentral.com/1472-6947/13/47/prepub
